# Exploring the multidimensional symptom experience in patients with inflammatory bowel disease—a contemporaneous network analysis

**DOI:** 10.3389/fmed.2025.1631207

**Published:** 2025-08-06

**Authors:** Yunzheng Di, Yamei Chen, Xiaoping Zhu, Rong Wang, Sijia Zhang, Pengcheng Sun

**Affiliations:** ^1^Department of Nursing, Shanghai Tenth People’s Hospital, Tongji University School of Medicine, Shanghai, China; ^2^School of Nursing, Anhui University of Chinese Medicine, Hefei, China; ^3^Gastrointestinal Endoscopy Department, Renji Hospital Affiliated to Shanghai Jiaotong University School of Medicine, Shanghai, China

**Keywords:** inflammatory bowel disease, network analysis, symptom management, symptom network, symptom network analysis

## Abstract

**Aim:**

To explore and visualize the relationships among multiple symptoms in patients with inflammatory bowel disease (IBD) and present empirical evidence for establishing personalized and precise symptom management strategies.

**Design:**

This is a quantitative research study conducted between May 2024 and March 2025 using a correlational research design.

**Methods:**

A total of 324 individuals diagnosed with IBD and hospitalized in Shanghai completed the Symptom Cluster Scale for Inflammatory Bowel Disease (SCS-IBD). We conducted multiple linear regression analysis to investigate factors related to the severity of overall IBD symptoms. After accounting for covariates, contemporaneous networks were constructed using all 18 symptoms.

**Results:**

It was determined that active IBD, years since IBD diagnosis, or those who have not received medication and surgery tend to have more severe IBD symptoms. Although fatigue was the most frequent (74.07%) and severe symptom (2.37 ± 1.161) in IBD, the strength centrality of fatigue was lower than that of weight loss and diarrhea. Weight loss (*r_s_* = 4.414, *r_scov_* = 5.202) and diarrhea (*r_s_* = 4.489, *r_scov_* = 5.109) are the core symptoms based on exhibiting the highest strength centrality values in both networks, regardless of whether covariates are included or not.

**Conclusion:**

Our findings identified that IBD experienced a heavy symptom burden of a severe nature, with weight loss and diarrhea being core symptoms, regardless of covariate adjustment.

## 1 Introduction

Inflammatory bowel disease (IBD), mainly including ulcerative colitis (UC) and Crohn’s disease (CD), is a class of chronic, recurrent, nonspecific intestinal inflammatory diseases with complex pathogenesis that involves the interactions of multiple factors such as genetics, environment, and immunity (1–3) Globally, the incidence and prevalence of IBD have been increasing year by year, and it has become a research hotspot and difficulty in the field of digestive system diseases. It is estimated that there are around 3 million adults affected with inflammatory bowel disease (either CD or ulcerative colitis) in the United States (4). In the Western world, the UC incidence rate ranges from 1 to 24 cases per 100,000 person-years, with the highest incidence rates observed in Scandinavia and Northern Europe (5). Prevalence is approximately 1 per 1,000.

The clinical manifestations of IBD are complex and varied, mainly manifested as abdominal pain, diarrhea, fatigue and weakness, malnutrition, sadness and depression, sleep disorders, and many other symptoms concurrently, which bring a heavy burden to the patients. The study found that nearly 75 percent of patients experienced at least two symptoms, with abdominal pain and diarrhea being the most prevalent and severe symptoms in IBD patients, followed by fatigue (6). In addition, the co-existence of symptoms such as malnutrition and depressed mood has also been reported. Therefore, effective identification and management of symptoms are essential to improve the quality of life for IBD patients (7).

Currently, there are few studies on IBD symptoms, and most of them focus on a single symptom in patients with IBD, failing to synthesize the associations between symptoms (8, 9). For example, Redeker et al. (10) found that patients with IBD experience different types and degrees of fatigue, which, in turn, interacts with the patient’s sleep quality and psychological state, leading to a vicious cycle of repeated. The European Society for Clinical Nutrition and Metabolism (ESPEN) guidelines note out that diarrhea symptoms in IBD are significantly more closely related to malnutrition symptoms (11). With the growing interest in studying IBD symptoms, symptom clusters are gradually being introduced into research to understand the associations between symptoms and their impact on patient outcomes. In 2005, Kim et al. (12) suggested that “symptom clusters consist of 2 or more related symptoms co-occurring, and the etiology and mechanism of occurrence of symptoms within the same symptom cluster may be different, but stable and independent of other symptom clusters.” Johansen et al. (13) investigated 573 IBD patients and found that there were three different symptom clusters in IBD patients: psychological, fatigue, and somatic. Recognizing symptom clusters is a commonly utilized scientific downscaling method that simplifies the complex relationships between symptoms. However, the process does not clearly articulate the mechanisms by which symptoms occur, and the interaction between symptoms remains ambiguous.

In recent years, symptom networks have been steadily applied to managing diseases, providing new insights for accurate symptom management. The concept of symptom network was proposed by Rubin and Zorumski (14) in 2015, explaining that the symptom network is a collective representation of the symptoms associated with a patient’s disease and that the core symptom is the one that plays a central role in the entire symptom group, and that the core symptom can be identified through network analysis methods, revealing the association and influence between different symptoms. The development of this concept not only reflects the interaction mechanism of symptoms in the real world, but also makes symptom management no longer limited to a single symptom or symptom group, but provides targets for precise intervention by identifying the core symptoms based on network node centrality (particularly strength), which is of great practical significance for improving the efficiency of symptom management and reducing the burden of symptoms on patients.

Earlier works have formulated symptom networks in multiple populations, with chemotherapy cancer patients being one of them, HIV-infected patients, and patients with mental disorders, to analyze the complex associations between various chronic symptoms (15–18). Despite previous research efforts, no studies have examined symptom networks related to the multidimensional symptoms experienced by patients with inflammatory bowel disease. The core symptoms of patients with long-term inflammatory bowel disease are not yet apparent when considered at the level of assessing mechanistic interplays between symptoms. This empirical groundwork is crucial for formulating precise, individualized approaches to symptom management. Consequently, this research aims to (1) explore the frequency, severity, and distress of IBD symptoms, (2) construct a symptom network of multidimensional symptom experience in patients with inflammatory bowel disease, and (3) unearth the core symptoms within the symptom networks.

## 2 Methods

### 2.1 Study design and settings

A cross-sectional was conducted at the Tenth People’s Hospital affiliated with Tongji University between May 2024 and January 2025. IBD patients were recruited from the Gastroenterology Clinic of this 50-bed hospital, which treats over 1,500 patients annually, ensuring an adequate sample size. Two specially trained research nurses within the department screened eligible participants. Following written informed consent, participants completed a printed questionnaire rating 18 symptoms on a severity scale, with an average completion time of 10 min. The Strengthening the Reporting of Observational Studies in Epidemiology (STROBE) checklist was followed to ensure the quality of the study (19). Ethical clearance was obtained from the Medical Ethics Committee of Tenth People’s Hospital (approval number: 25KN223).

### 2.2 Participants

Participants were included based on the following criteria: (1) Patients diagnosed as having IBD. (2) Age: ≥18 years old. (3) Provided informed consent. Participants were excluded based on: (1) acute severe illness compromising survey cooperation and (2) documented history of psychiatric disorders or communication disabilities. All enrolled participants provided voluntary, written informed consent. During data collection, research nurses immediately checked the completeness of the questionnaire. Questionnaires with missing items in the Symptom Cluster Scale for Inflammatory Bowel Disease (SCS-IBD) scale were either completed on-site or excluded if participants declined to provide missing data. Of 351 questionnaires initially collected, 27 (7.7%) were excluded due to incomplete responses (>10% missing items), resulting in 324 complete cases for analysis.

### 2.3 Sample size estimation

This study included 18 symptomatic variables and three covariates; the three covariates were stage of IBD, years since IBD diagnosis, and the type of treatment received. For a 21-node partial correlation network, the total number of correlation parameters was calculated using the formula [*n* + *n*(*n* – 1)/2], where *n* represents the number of nodes. Applying this formula to the 21 nodes yielded 231 unique partial correlation parameters, necessitating a minimum sample size of 231 participants to achieve statistical validity (20). The present study included 324 cases, exceeding the required threshold for robust network analysis.

### 2.4 Measures

#### 2.4.1 Sociodemographic and clinical data

Sociodemographic data were collected using a custom-developed questionnaire validated through pilot testing. This instrument captured detailed participant characteristics, including (1) demographic variables: age, gender, education level, marital status, employment status, medical insurance type, and primary caregiver relationship and (2) clinical variables: type of IBD, stage of IBD, years since IBD diagnosis, and the type of treatment received. Staging was based on independent assessment by two gastroenterologists. The type of treatment received was according to the primary intervention during the 6 months before enrollment. Pharmacological treatment included 5-aminosalicylic acid, immunosuppressants, or biologics alone or in combination, and surgical treatment was defined as a previous resection of an intestinal segment or fistula.

#### 2.4.2 Symptoms of patients with IBD

To assess the symptoms experienced by IBD patients, we utilized the Symptom Cluster Scale for Inflammatory Bowel Disease (SCS-IBD) (21). We chose this validated tool because it provides a holistic evaluation of IBD-related symptoms experienced by patients during the preceding 4 weeks. The scale was assessed in terms of frequency of symptoms, severity, and distress. The SCS-IBD consists of 5 symptom clusters and 18 symptoms, including abdominal symptom clusters (diarrhea and abdominal pain), intestinal symptom clusters (abdominal distension, bloody purulent stool, tenesmus, perianal abscess, anal fissure, and anal fistula), nutritional symptom clusters (nutritional deficiencies, weight loss, and anemia), systemic symptom clusters (skin lesions, oral mucosal lesions, and ocular lesions), and psychosomatic symptom clusters (fatigue, anxiety, depression, and disturbed sleep). Symptom severity was rated using a 5-point Likert scale (1 = not at all, 5 = very severely). An individual symptom score was calculated for descriptive analyses as the mean of its frequency, severity, and distress ratings. The severity dimension alone was used for inferential analyses (e.g., regression). It is an 18-item instrument with a symptom domain scoring range of 54–270. In the current sample, the scale demonstrated excellent internal reliability (Cronbach’s α = 0.910).

### 2.5 Data analysis

Statistical analyses were performed using R version 4.4.0 ^1^. Missing data handling: Given the real-time quality control during data collection, the final analytical dataset contained no missing values across all 18 symptoms and covariates. Thus, complete case analysis was employed without requiring imputation methods. Demographic characteristics and symptom metrics (prevalence, severity, and distress) were described using (1) categorical variables: frequencies (*n*) and percentages (%) and (2) continuous variables: means [standard deviation (SD)], medians (interquartile range (IQR): P25–P75). Multivariable linear regression was employed to identify factors associated with overall IBD symptom severity. Before performing linear regression analyses, we conduct linearity assumption tests and homogeneity of variance tests using residual scatter plots, normality tests using residual histograms, and finally multicollinearity tests of predictor variables using variance inflation factor (VIF) values.

To characterize the complex interactions between symptoms in the 18 SCS-IBD measures, we employed network analysis based on principles from psychological and chronic disease research (22). Quantifying pairwise associations between symptoms using Spearman’s correlation to account for the non-normal distributions common to symptom severity scales (23). We applied the EBICglasso algorithm to minimize spurious connections, which penalizes weak edges while preserving clinically meaningful interactions (24). Network density is defined as the proportion of possible edges that are present (i.e., non-zero) in the estimated network. Covariates that demonstrated significant associations with overall symptom severity were incorporated into the network analysis to estimate partial correlations among the 18 symptoms, controlling for confounding variables (25). Networks were visualized using the Fruchterman–Reingold algorithm, positioning core symptoms closer to the network core (26).

In the symptom network, node centrality can reflect the importance of a symptom; this study mainly assessed the core symptom in terms of strength, closeness, and betweenness (27). Strength refers to the sum of the absolute value of the edge weight between a node and all the nodes that are directly connected to it, and the higher the intensity centrality, it indicates that the more likely the symptom is to occur at the same time as the other symptoms, and the more powerful its influence is within the whole network; “Closeness” refers to the inverse of the average distance between a node and other directly connected nodes; “Betweenness” refers to the number of times a node acts as an intermediary in the entire network (27). Symptoms exhibiting the highest values in the strength centrality metric were designated as the core symptoms in the network, as strength centrality is the most critical indicator representing core symptoms in prior research (24, 28). Symptoms exhibiting the highest values in multiple node centrality (strength, closeness, and betweenness) were designated as core symptoms. The stability of these centrality estimates was evaluated using the correlation stability (CS) coefficient, with interpretive thresholds of ≥0.25 (minimal stability) and ≥0.5 (strong stability) (24). Furthermore, node-specific predictability was computed using mixed graphical modeling (MGM) to estimate determinacy coefficient (R^2^) for each variable derived from its covarying neighbors. Node predictability (R^2^) measures the proportion of a symptom’s variance explained by its directly connected neighbors. We adopted empirically established predictability thresholds: *R*^2^ ≥ 0.65 indicates high network-embeddedness (where interactions within the symptom network primarily drive symptoms), while *R*^2^ ≤ 0.30 suggests prominent influences from unmeasured external factors (e.g., biological variables and environmental triggers) (29, 30). These thresholds align with conventions in clinical network analysis (18, 26).

## 3 Results

### 3.1 Characteristics of participants

A total of 324 participants were included in this study, and their characteristics are presented in Table 1. The average age of the participants was 43 ± 14.75. Majority of the participants were male (*n* = 220, 67.9%), were married (*n* = 252, 77.8%), had a university or above education level (*n* = 192, 59.35%), had other employment types (*n* = 160, 49.4%), with medical insurance (*n* = 312, 96.3%), whose primary caregiver is spouse (*n* = 144, 44.4%), had more Crohn’s disease (*n* = 228, 70.4%). The staging of the disease is primarily active (*n* = 188, 58.0%). The average years since IBD diagnosis was 5.36 ± 5.77 years. In terms of treatment modalities, medication was the primary therapy received by the majority of participants (*n* = 176, 54.3%), followed by medication and surgery (*n* = 80, 24.7%), and otherwise (*n* = 52, 16%).

**Table 1:** Characteristics of participants (*n* = 324).

Characteristics	*N* (%), Mean ± SD, M (P25, P75)
Age	43.22 ± 14.75, 43 (30, 55)
Gender	
Male	220 (67.9)
Female	104 (32.1)
Education level	
Primary school or below	36 (11.1)
Secondary school	96 (29.6)
University or above	192 (59.3)
Marital status	
Single	72 (22.2)
Married	252 (77.8)
Employment	
Yes, full-time job	144 (44.4)
Yes, part-time job	20 (6.2)
Otherwise	160 (49.4)
Medical insurance	
Without medical insurance	12 (3.7)
With medical insurance	312 (96.3)
Primary caregiver	
Self	104 (32.1)
Spouse	144 (44.4)
Parents	60 (18.5)
Children	16 (4.9)
Type of IBD	
Crohn’s disease	228 (70.4)
Ulcerative colitis	96 (29.6)
Stage of IBD	
Active stage	188 (58.0)
Remission	136 (42.0)
Years since IBD diagnosis	5.36 ± 5.77, 4 (1, 7)
Type of treatment received	
Medication	176 (54.3)
Surgery	16 (4.9)
Both medication and surgery	80 (24.7)
Otherwise*	52 (16)

Data presented as *n* (%) for categorical variables (shows proportional distribution), mean ± standard deviation (SD, quantifies average value and variability), and median (interquartile range, IQR: P25, P75, and robust center and spread measure for skewed data) for continuous variables. IBD, inflammatory bowel disease; CD, Crohn’s disease; UC, ulcerative colitis. *For employment—includes unemployed, retired, students, etc.; For Treatment:—includes no treatment and complementary or alternative therapies; Primary caregiver is self—patient primarily manages their care. Two independent gastroenterologists assessed disease stage (active/remission). Years since diagnosis: Time elapsed since initial IBD diagnosis.

### 3.2 Symptom prevalence, severity, and distress

Fatigue (74.07%) was the most common symptom, followed by disturbed sleep (67.90%) and diarrhea (66.8%). The most distressing symptoms were diarrhea (2.40 ± 1.396) and fatigue (2.37 ± 1.234), with disturbed sleep (2.31 ± 1.275) closely trailing. Regarding severity, fatigue (2.37 ± 1.161) led, followed by disturbed sleep (2.2 ± 1.199) and diarrhea (2.22 ± 1.209), as detailed in Table 2.

**Table 2:** Symptom prevalence, severity, and distress of participants.

Symptoms	*N* (%)	Symptom severity		Symptom distress	
		Mean (SD)	*M* (P25, P75)	Mean (SD)	*M* (P25, P75)
Diarrhea	217 (66.8)	2.22 (1.209)	2 (1, 3)	2.40 (1.396)	2 (1, 3)
Abdominal pain	188 (58.02)	1.98 (1.113)	2 (1, 3)	2.07 (1.276)	2 (1, 3)
Abdominal distension	152 (46.91)	1.64 (0.837)	1(1, 2)	1.69 (0.991)	1 (1, 2)
Bloody purulent stool	144 (44.44)	1.81 (1.158)	1(1, 2)	1.83 (1.227)	1 (1, 2)
Tenesmus	200 (61.73)	2.02 (1.079)	2 (1, 3)	2.00 (1.113)	2 (1, 3)
Perianal abscess	36 (11.11)	1.15 (0.525)	1 (1, 1)	1.12 (0.456)	1 (1, 1)
Anal fissure	20 (6.17)	1.09 (0.392)	1 (1, 1)	1.11 (0.522)	1 (1, 1)
Anal fistula	48 (14.81)	1.37 (0.963)	1 (1, 1)	1.35 (0.959)	1 (1, 1)
Nutritional deficiencies	204 (62.96)	2.11 (1.124)	2 (1, 3)	2.12 (1.253)	2 (1, 3)
Weight loss	180 (55.56)	2.17 (1.305)	2 (1, 3)	2.10 (1.332)	2 (1, 3)
Anemia	132 (40.74)	1.58 (0.942)	1(1, 2)	1.56 (0.944)	1(1, 2)
Skin lesions	36 (11.11)	1.21 (0.624)	1 (1, 1)	1.16 (0.509)	1 (1, 1)
Oral mucosal lesions	52 (16.05)	1.20 (0.483)	1 (1, 1)	1.20 (0.555)	1 (1, 1)
Ocular lesions	36 (11.11)	1.12 (0.365)	1 (1, 1)	1.14 (0.438)	1 (1, 1)
Fatigue	240 (74.07)	2.37 (1.161)	2 (1, 3)	2.37 (1.234)	2 (1, 3)
Anxiety	168 (51.86)	1.94 (1.117)	2 (1, 3)	1.96 (1.129)	2 (1, 3)
Depression	80 (24.69)	1.43 (0.889)	1 (1, 1)	1.48 (0.972)	1 (1, 1)
Disturbed sleep	220 (67.90)	2.27 (1.199)	2 (1, 3)	2.31 (1.275)	2 (1, 3)

Severity/distress: 5-point Likert scale (1 = minimal, 5 = extreme) → higher scores = greater burden. Median (IQR): Reported where distributions are skewed (e.g., perianal symptoms). Fatigue is both the most prevalent (74.07%) and the most severe symptom. Diarrhea: Highest distress (2.40) despite lower prevalence than fatigue. Psychological symptoms: Anxiety (51.86%) > depression (24.69%) → Mental health burden focus. SD: Higher values (e.g., diarrhea distress SD = 1.396) → Greater interpatient variability IQR (P25–P75): Interquartile range → Middle 50% of responses (robust to outliers). SD, standard deviation; IQR, interquartile range, P25, 25th percentile; P75, 75th percentile.

### 3.3 Associated factors with the overall symptom severity

Table 3 shows the linear regression models for overall symptom severity. All key linear regression assumptions (linearity, homoscedasticity, absence of multicollinearity, and normality of residuals) were tested and found to be satisfied. To ensure a higher level of reliability and rigor of the findings, a more stringent *p <* 0.001 was used as the level of significance in this study instead of the commonly used *p <* 0.05 to reduce the possibility of false-positive results and to make the conclusions more convincing. The active stage (β = 0.496, *p <* 0.0001), years since IBD diagnosis (β = 0.265, *p <* 0.0001), the type of treatment received is otherwise (β = 0.139, *p* = 0.008) demonstrated statistical significance (*p <* 0.05) in the linear regression model were incorporated as covariates in the partial correlation network analysis. A *post hoc* power analysis for the multivariable linear regression model was performed using G*Power 3.1. With 13 predictors, a medium effect size (Crohn’s *f*^2^ = 0.15), α = 0.001, and *N* = 324, the achieved power was 0.98, indicating sufficient statistical power to detect significant effects.

**Table 3:** Linear regression model of total symptom severity scores.

Characteristics	*B*	β	*p*	95.0% CI for *B*		VIF
				Lower bound	Upper bound	
Age	0.017	0.032	0.611	−0.049	0.084	2.086
University or above (vs. otherwise)	−1.228	−0.108	0.055	−2.398	−0.058	1.409
Male (vs. female)	0.359	0.064	0.191	−0.537	2.678	1.409
Married (vs. otherwise)	1.071	−0.102	0.085	−4.088	0.267	1.229
Full-time job (vs. otherwise)	−1.911	0.063	0.202	−0.532	2.510	1.788
With medical insurance (vs. otherwise)	0.989	−0.123	0.011	−8.939	−1.194	1.246
Primary caregiver is self (vs. otherwise)	−5.067	0.107	0.025	0.226	3.365	1.167
Crohn’s disease (vs. ulcerative colitis)	1.796	−0.121	0.038	−4.012	−0.113	1.171
Active stage (vs. remission)	−2.063	0.496	*<* **0.001** ^a^	6.303	9.383	1.729
Years since IBD diagnosis	7.843	0.265	*<* **0.001** ^a^	0.220	0.498	1.261
Surgery (vs. medication)	−1.817	−0.050	0.308	−5.316	1.683	1.255
Surgery and medication (vs. medication)	−0.730	−0.040	0.452	−2.636	1.176	1.473
Otherwise* (vs. medication)	2.954	0.139	< 0.008^a^	0.773	5.136	1.399

B: Unstandardized coefficient → Unit change in severity per predictor change. β: Standardized coefficient → Effect size comparison (β = 0.496 for active disease = strongest) *p* < 0.001 threshold: Reduced false-positive risk vs conventional *p* < 0.05. VIF < 5: No multicollinearity concerns (all VIF < 2.1). Key clinical drivers: Active disease stage: +49.6% severity (*p* < 0.0001). Longer diagnosis duration: +26.5% severity per year (*p* < 0.0001). Non-standard treatments: +13.9% severity (*p* = 0.008). Statistical validation: Adjusted *R*^2^ = 0.372*: 37.2% symptom variance explained. *Post hoc* power = 0.98*: Adequate detection of medium effects (*f*^2^ = 0.15). Residual diagnostics: Confirmed linearity/homoscedasticity/normality (Supplementary Figures S1, S2). B, unstandardized coefficient; *β*, standardized coefficient; VIF, variance inflation factor; CI, confidence interval; UC, ulcerative colitis. ^a^Bolded P-values indicate statistically significant predictors of the overall severity outcome (*P* < 0.01). These significant variables were selected for inclusion in the subsequent network analysis.

### 3.4 Network estimation and centrality indices of IBD symptoms

**Figure 1:**
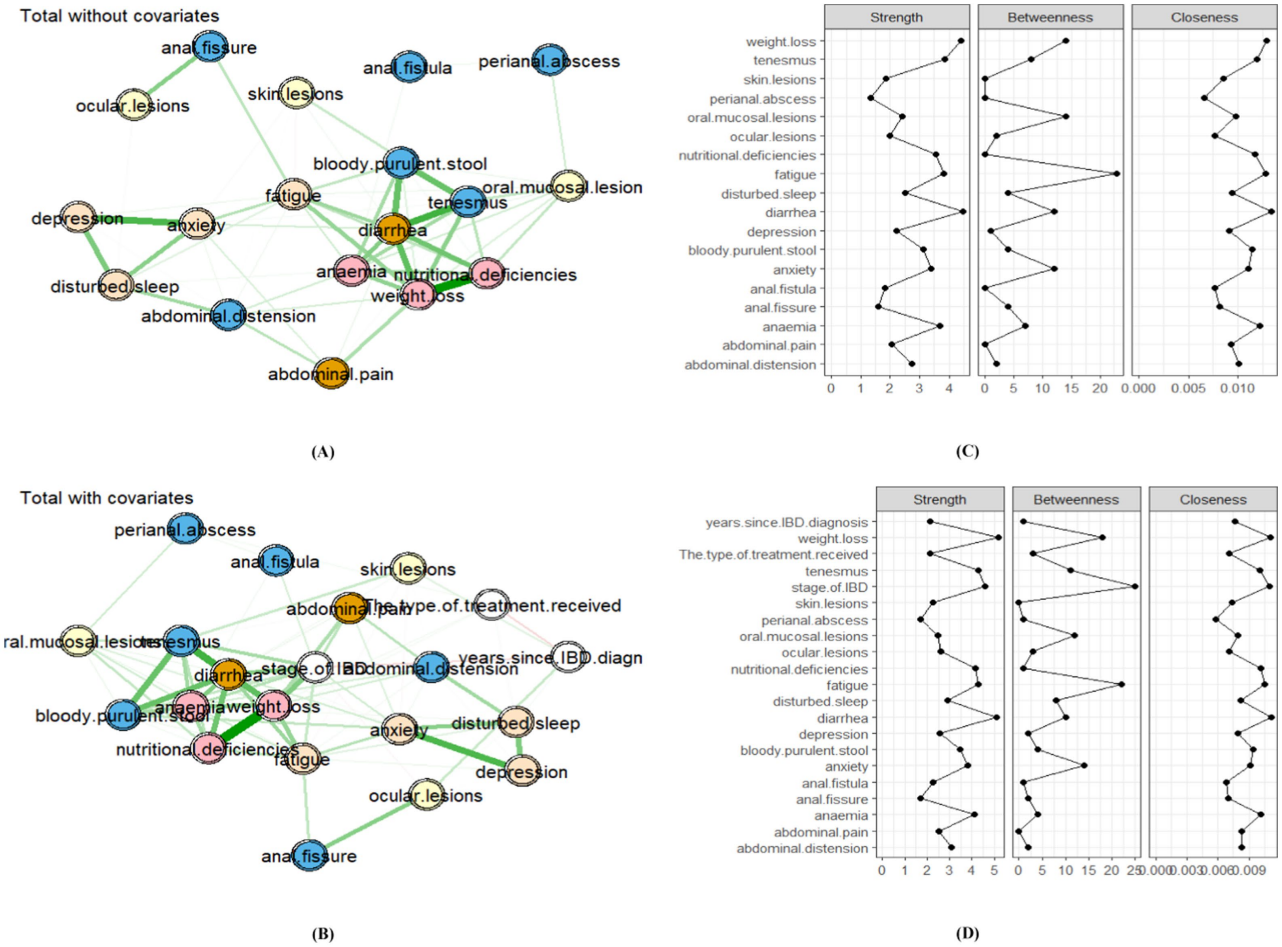
Symptom networks and centrality indices in patients with inflammatory bowel disease (IBD) (*N* = 324). **(A)** Unadjusted symptom network: Partial correlation network of the 18 IBD symptoms without covariate adjustment. Nodes represent symptoms. Edges represent regularized partial correlations (edge thickness and saturation indicate strength; green: positive; red: negative). Numbers within nodes indicate node predictability (R^2^: proportion of variance explained by neighboring symptoms). The Fruchter–Man–Reingold layout places nodes with higher centrality closer to the center, and central nodes have higher strength centrality. **(B)** Covariate-adjusted symptom network: Partial correlation network adjusting for significant covariates associated with total symptom severity (Active disease stage, Years since diagnosis, Treatment type “Otherwise”). The layout and edge representation are exactly as in subpart **(A). (C)** Centrality indices for the unadjusted network: Standardized centrality measures (Strength, Closeness, and Betweenness) for each symptom in the unadjusted network **(A)**. Symptoms are ordered by strength centrality. **(D)** Centrality indices for the adjusted network: Standardized centrality measures for each symptom in the covariate-adjusted network **(B)**. Order and abbreviations are the same as in subpart **(C)**. **(C,D)** Centrality indices: Strength: Sum of absolute edge weights (higher = more network influence). Closeness: Inverse of average shortest path to other nodes. Betweenness: Frequency of acting as a bridge between symptoms.

**Figure 2:**
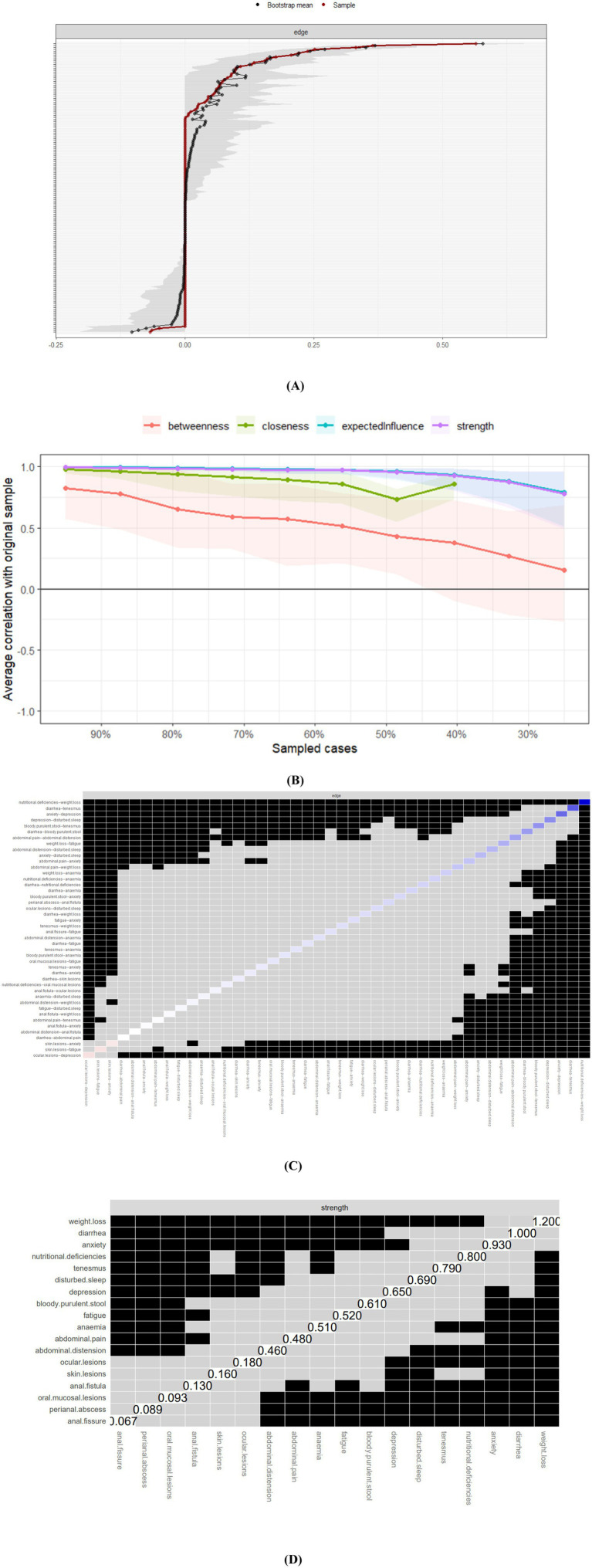
Accuracy, stability, and bootstrapped difference tests for the unadjusted IBD symptom network (*N* = 324). **(A)** Edge weight accuracy: Nonparametric bootstrap 95% confidence intervals (CIs; gray areas) for each edge weight in the unadjusted network (Figure 1A). The red dot indicates the original edge weight estimate. Edge order corresponds to the strength of the original estimate (strongest to weakest). Gray bands = 95% bootstrap confidence intervals (CIs); Red dot = original edge weight. **(B)** Centrality stability: Correlation Stability (CS) coefficients for Strength, Expected Influence (EI), and Closeness centrality indices. The dashed line indicates the recommended threshold for acceptable stability (CS ≥ 0.25). The dotted line indicates strong stability (CS ≥ 0.5). CS-coefficient > 0.5 = high stability (dotted line). **(C)** Edge weight differences: Results of bootstrapped difference tests comparing edge weights. Cells are colored if the difference between the row’s and column’s edges is significant (*p* < 0.05, uncorrected). Darker blue indicates larger differences. The most potent edge (WL-ND: Weight Loss-Nutritional Deficiency) is highlighted. Blue cells = significant edge weight difference (*p* < 0.05, uncorrected). Darker blue = larger difference. **(D)** Node centrality differences: Results of bootstrapped difference tests evaluating node centrality (Strength). Cells are colored if the difference in centrality between the node in the row and the node in the column is significant (*p* < 0.05, uncorrected). Cells are colored if strength centrality differs significantly (*p* < 0.05).

Partial correlation networks of the whole cohort (*N* = 324), with and without covariate control, were displayed in Figure 1, alongside standardized centrality measures. The density of the unadjusted network (Figure 1A) was 55.56% (85 significant edges out of 153 possible), while the density of the covariate-adjusted network (Figure 1B) was 54.29% (114 edges out of 210 possible). The strongest unadjusted connection was observed between nutritional deficiency and weight loss (*r* = 0.56), with confidence intervals surpassing all other edges (Figure 2C). This association remained the strongest in the adjusted network. Edge weights were provided in Supplementary Table S1 (unadjusted) and Supplementary Table S2 (adjusted). Node predictability values in the network without covariates ranged from 21.2 to 78.1%. Notably, weight loss and diarrhea showed the highest predictability, with 78.1 and 69.2% of their variance explained by their neighboring symptoms. Weight loss (*r_s_* = 4.414, *r_scov_* = 5.202) and diarrhea (*r_s_* = 4.489, *r_scov_* = 5.109) exhibited the highest strength centrality values and were thus identified as the core symptoms in both networks. Detailed centrality measures for both networks were presented in Supplementary Table S3 (unadjusted) and Supplementary Table S4 (adjusted).

### 3.5 Accuracy, stability, and bootstrapped difference test for edges and nodes

**Figure 3:**
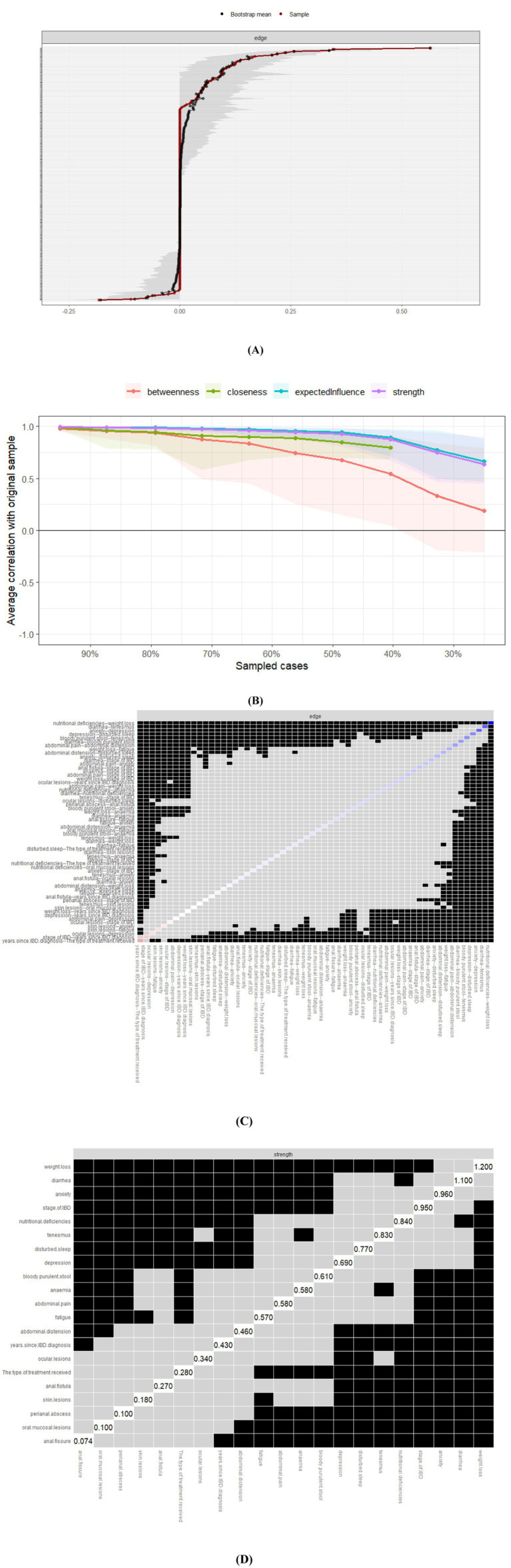
Accuracy, stability, and bootstrapped difference tests for the covariate-adjusted IBD symptom network (*N* = 324). **(A)** Edge weight accuracy: Nonparametric bootstrap 95% confidence intervals (CIs; gray areas) for each edge weight in the covariate-adjusted network (Figure 1B). The red dot indicates the original edge weight estimate. Edge order corresponds to the strength of the original estimate (strongest to weakest). Gray bands = 95% bootstrap confidence intervals (CIs); Red dot = original edge weight. **(B)** Centrality stability: Correlation Stability (CS) coefficients for Strength, Expected Influence (EI), and Closeness centrality indices in the adjusted network. Dashed (CS ≥ 0.25) and dotted (CS ≥ 0.5) lines as in Figure 2B. CS-coefficient > 0.5 = high stability (dotted line). **(C)** Edge weight differences: Results of bootstrapped difference tests comparing edge weights in the adjusted network. Interpretation as in Figure 2C. The most potent edge (WL-ND: weight loss–nutritional deficiency) is highlighted. Blue cells = significant edge weight difference (*p* < 0.05, uncorrected). Darker blue = larger difference. **(D)** Node centrality differences: Results of bootstrapped difference tests evaluating node centrality (Strength) in the adjusted network—interpretation as in Figure 2D. WL (Weight Loss) shows the highest distinctiveness. Cells are colored if strength centrality differs significantly (*p* < 0.05).

Tight bootstrap confidence intervals confirmed the accuracy of both unadjusted and covariate-adjusted symptom networks (Figures 2A, 3A). Subset bootstrap analysis revealed that the correlation stability coefficients (CS coefficients) of both expected influence and strength centrality are greater than 0.5, indicating that the network exhibits high robustness (24) (Figures 2B, 3B). Bootstrap edge difference tests (Figures 2C, 3C) indicated that the most potent edge (“weight loss–diarrhea”) differed significantly from 95% of other edges in both models. Node difference tests (Figures 2D, 3D) showed weight loss had the highest distinctiveness (DTs = 1.2) regardless of covariate adjustment.

## 4 Discussion

In this study, we constructed for the first time a network of IBD contemporaneous symptoms through five dimensions of SCS-IBD, including abdominal symptom clusters, intestinal symptom clusters, nutritional symptom clusters, systemic symptom clusters, and psychosocial symptom clusters. The topological examination of contemporaneous symptom networks permits researchers to delineate core symptomatic elements through network centrality metrics, empowering clinicians to formulate mechanism-driven treatment strategies based on quantified intersymptom connectivity patterns. In this study, we found that although fatigue was the most frequent and severe symptom of IBD, the strength centrality of fatigue was not the highest. The stage, years of diagnosis, and treatment received for IBD are associated with increased severity of IBD. In addition, weight loss and diarrhea are the core symptoms in the network, regardless of the presence of covariates.

This study suggests that patients with active IBD, years since IBD diagnosis, or those who have not received medication and surgery tend to have more severe IBD symptoms. IBD influences, excluding the disease itself, have been reported to negatively affect patients’ health-related quality of life, including psychological (31), financial burden (32), and sleep disturbances (33). Identifying factors associated with severe symptoms of IBD can help healthcare providers target people at high risk for developing severe symptoms. Previous studies have shown that symptoms are more severe in active IBD than in remission; pro-inflammatory cytokines are significantly elevated in the intestinal mucosa during active IBD, driving neutrophil and T-cell infiltration, leading to epithelial damage, crypt abscesses, and barrier dysfunction, which triggers abdominal pain, diarrhea, and bleeding (34). In addition, inflammation-related neurosensitization and intestinal dyskinesia further exacerbate symptoms (35). Patients with IBD who have not received medications or surgery often present with persistent or fluctuating moderate-to-severe symptoms, including bloody stools and weight loss, and are at increased risk of complications due to chronic inflammation leading to progressive mucosal damage and fibrosis (36). Lack of immunosuppressive intervention allows for an uncontrolled inflammatory cascade response and sustained activation of pro-fibrotic factors, accelerating structural destruction of the intestinal tract (35). We also found a relationship between years since IBD diagnosis and the severity of symptoms associated with IBD. Ocular lesions in patients with IBD show a time-dose-dependent relationship with disease duration, with those with CD, long-term active disease, and frequent relapses being at the highest risk in particular. Troncoso et al. (37) noted that in patients with IBD with a long duration of the disease, ocular lesions, such as uveitis, have an insidious onset and prolonged duration of illness, and are more symptomatic, and in severe cases, may lead to blindness; scleral episcleritis is often associated with intestinal inflammatory activity, and the longer the duration of the disease, the more frequent the episodes may be, etc.

Although previous studies have reported a high prevalence of fatigue in IBD patients of up to 48.6% (76.3% in active vs. 32.7% in remission) (38), the symptom network analysis in the present study showed that fatigue was a lower strength centrality than weight loss and diarrhea, both with and without covariates. Our results suggest that fatigue may not be a core symptom of IBD, despite its high prevalence and severity. It may be that fatigue is only weakly correlated with systemic inflammatory markers (39), whereas core intestinal symptoms directly reflect localized lesion activity. Fatigue exhibits a high degree of strength centrality in cross-disease studies (40, 41), but is moderated by organ-system symptom interactions in IBD-specific networks. Node centrality (particularly strength) indicates identifying core symptoms from a mechanistic perspective. Traditional paradigms tend to equate high-frequency, high-intensity clinical symptoms as key intervention targets (42, 43). However, studies similar to the one by Zhu et al. (18) confirmed through network analysis methods that high-frequency or high-intensity symptoms may exist only as sentinel symptoms, and their network influence is lower than that of core symptoms highlighted by centrality indicators. Based on the network analysis framework, Strength centrality, as a key metric of node centrality, can quantify the regulatory weight within the system and identify the pivotal nodes (core symptoms defined by high strength centrality)” that truly drive the deterioration of symptom clusters; precise interventions targeting symptoms with high and medium strength centrality can generate network cascade effects by modulating the core pathological pathways, which can in turn achieve exponential enhancement of the efficacy of symptom management (44). Therefore, clinical practice must transcend traditional assessment dimensions and construct a network centrality-oriented IBD symptom management system.

Network analysis in this study revealed that weight loss (*r_s_* = 4.414, *r_scov_* = 5.202) and diarrhea (*r_s_* = 4.489, *r_scov_* = 5.109) constitute the core nodes of the IBD symptom network. This identification of core symptoms based on strength centrality demonstrated robustness to including three covariates in the network model. While this identification is based on their pivotal role in symptom network connectivity, it does not diminish the clinical significance of high-prevalence or high-distress symptoms such as fatigue or bloody stools. Instead, centrality metrics provide a complementary lens to traditional prioritization by revealing symptoms that may be leverage points for systemic intervention. For example, targeting a highly central symptom like weight loss could disrupt pathological cycles (e.g., malnutrition → barrier dysfunction → inflammation) and yield cascading benefits across interconnected symptoms, whereas isolated management of high-frequency symptoms may have limited network-wide impact. The high predictability of weight loss (78.1%) and diarrhea (69.2%) indicates that their severity is primarily governed by interactions with co-occurring symptoms (e.g., nutritional deficiency and abdominal pain interactions). This mechanistic pattern supports clinical strategies that simultaneously target multiple connected symptoms—such as combining low-FODMAP diets (for diarrhea) with protein-calorie supplementation (for weight loss) (11) To the best of our knowledge, this represents one of the first investigations into symptoms in patients with IBD using network analysis, providing novel insights into the structural role of core symptoms within the symptom network (45). It is suggested that early identification of signs of worsening bowel symptoms with dynamic nutritional assessment should be a priority for clinical monitoring in the long-term management of IBD. Previous studies have confirmed that the symptom burden experienced by IBD patients is multidimensional, encompassing pathological processes such as altered intestinal permeability, impaired nutrient absorption, metabolic disorders, and immune activation (46–48). Contrary to being merely a passive consequence of disease severity, weight loss exhibits the highest strength centrality in our network, suggesting it may actively propagate dysfunction within the symptom system. This aligns with its known biological role: weight loss in IBD reflects malnutrition and correlates with the severity of mucosal inflammation (49–51), creating a self-perpetuating cycle (e.g., impaired barrier function → malabsorption → weight loss → exacerbated inflammation). Targeting weight loss through nutritional support may thus disrupt this cycle at its hub, offering broader therapeutic leverage than managing downstream symptoms alone (49–51). Similarly, diarrhea—a known marker of disease activity (34, 36)—occupies a central position, underscoring its dual role as both a direct indicator of pathological severity and a hub amplifying systemic dysfunction. This convergence of network centrality and clinical biomarkers supports the translational relevance of our findings.

The gut-system symptom interaction presents a dynamic centrality in the IBD disease process. With prolonged disease duration, patients may experience a pathological transition from osmotic diarrhea during acute exacerbations to malabsorption during remission, which directly affects treatment response and quality of life. Data from longitudinal cohort studies have shown that weight loss lasting more than 6 months increases the risk of hospitalization by 2.3-fold in patients with IBD and significantly reduces the response rate to biologic therapy (52–54). Therefore, there is an urgent need for clinical prognostic models that include symptom network parameters to guide individualized treatment decisions.

Intervention strategies targeting core symptoms (i.e., those with highest strength centrality) should integrate multidimensional pathways: (1) nutritional modification based on a low-FODMAP diet reduces the frequency of diarrheal episodes by 73% (55); (2) anti-TNFα agents in combination with nutritional support increase the rate of attained weight regain in moderate-to-severe patients up to 68% within 6 months (56); and (3) microbiota-targeted therapies improve diarrheal symptoms (both by modulating the functioning of the gut-brain axis) simultaneously with body composition indicators (57). symptoms and body composition indicators (57). These interventions must precisely match the disease phenotype, inflammatory activity, and complication risk stratification.

### 4.1 Limitations and future directions

This study has several limitations that should be acknowledged to inform future research and clinical interpretation. First, the cross-sectional design inherently limits the ability to make causal inferences. Contemporaneous network analysis identifies statistical associations between co-occurring symptoms but cannot determine temporal precedence or causal direction (e.g., whether weight loss exacerbates inflammation or vice versa). Thus, mechanistic interpretations of symptom interactions (e.g., “core symptoms drive the network”) remain hypothetical and require longitudinal validation. While centrality metrics can identify statistically influential symptoms, they do not imply causality. Longitudinal or dynamic network models are needed to understand symptom progression and causally test intervention targets. Second, all symptom data were collected via the self-reported SCS-IBD, without inclusion of objective biological or clinical markers such as CRP, fecal calprotectin, hemoglobin, or sleep indices. This limits the interpretation of symptoms like fatigue, which often have physiological and psychological contributors. Additional studies should focus on integrate biomarkers and clinical assessments to objectively verify and contextualize subjective symptom data, thereby enhancing the understanding of these symptoms. Third, although fatigue showed relatively low strength centrality, it was one of the most prevalent and distressing symptoms. Without accounting for causes such as anemia, inflammation, or depression, the model may underestimate its clinical importance. Fatigue should be interpreted cautiously, and future studies should include multidimensional data to assess its etiology and systemic impact. Fourth, our analysis did not assess or stratify patients by objective remission status or mucosal healing, which are now standard therapeutic endpoints in IBD management. Symptom improvement alone does not equate to disease control, and future symptom network models should incorporate endoscopic and biochemical measures to align with current treat-to-target strategies. Fifth, although our total sample size (*N* = 324) was adequate for network estimation, it was insufficient for subgroup comparisons by gender, age, or treatment modality. Larger, stratified samples are needed to explore whether symptom networks differ across clinical subpopulations. Sixth, we did not stratify by IBD phenotype (Crohn’s disease vs. ulcerative colitis) or extent/severity of disease. While this provided a unified symptom network, it may mask phenotype-specific dynamics. Future studies should construct phenotype-stratified and severity-stratified networks to tailor symptom management more precisely. Seventh, this was a single-center study conducted at a tertiary hospital in Shanghai, which may limit its generalizability to other settings or patient populations. Multicenter or population-based research would improve the external validity of network-informed findings. Eighth, centrality metrics should not be viewed as stand-alone clinical decision tools. Symptoms such as abdominal pain or rectal bleeding, though less central in the network, may carry high clinical urgency or patient distress. Centrality must be interpreted alongside patient-reported outcomes (PROs), clinical severity, and patient priorities, rather than as a substitute for clinical judgment. Future research should prioritize (1) using time-series data to map symptom trajectories and identify critical intervention windows, (2) investigating how biological factors mediate symptom interactions, and (3) targeting core symptoms to test whether alleviating these hubs disrupts detrimental symptom cycles.

## 5 Conclusion

Our findings identified a symptom network of multidimensional symptom experience in 324 patients with IBD, with weight loss and diarrhea consistently exhibit the highest strength centrality and are thus the core symptoms in the symptom network adjusted or unadjusted for clinical covariates, identifying them as top-priority targets for clinical management due to their strong statistical interconnectivity with other symptoms. Future mechanistic studies should test whether targeting these nodes disrupts symptom networks. This study provides novel insights into the complex symptom interplay in IBD through network analysis. Identifying weight loss and diarrhea as core symptoms based on strength centrality underscores their clinical priority in personalized care. These findings lay the groundwork for precision nursing strategies that leverage network metrics to optimize symptom control and improve quality of life for IBD patients.

## Implications for the profession and/or patient care

Healthcare professionals should prioritize managing IBD’s core symptoms—weight loss and diarrhea—via tailored dietary and medication strategies, disrupting symptom interactions to improve patients’ quality of life and recovery.

## Patient or public involvement

All participants were from the gastroenterology department of a single hospital.

## Data Availability

The raw data supporting the conclusions of this article will be made available by the authors, without undue reservation.
