# Correction: Exploring the multidimensional symptom experience in patients with inflammatory bowel disease—a contemporaneous network analysis

**DOI:** 10.3389/fmed.2025.1686171

**Published:** 2025-09-02

**Authors:** Yunzheng Di, Yamei Chen, Xiaoping Zhu, Rong Wang, Sijia Zhang, Pengcheng Sun

**Affiliations:** ^1^Department of Nursing, Shanghai Tenth People's Hospital, Tongji University School of Medicine, Shanghai, China; ^2^School of Nursing, Anhui University of Chinese Medicine, Hefei, China; ^3^Gastrointestinal Endoscopy Department, Renji Hospital Affiliated to Shanghai Jiaotong University School of Medicine, Shanghai, China

**Keywords:** inflammatory bowel disease, network analysis, symptom management, symptom network, symptom network analysis

The order of authors in the author list of the published paper was erroneously given as:

Yunzheng Di, Xiaoping Zhu, Yamei Chen, Rong Wang, Sijia Zhang, Pengcheng Sun.

The correct author list reads:

Yunzheng Di, Yamei Chen, Xiaoping Zhu, Rong Wang, Sijia Zhang, Pengcheng Sun.

The original version of this article has been updated.

