# Supplementary material for: Exploring the multidimensional symptom experience in patients with inflammatory bowel disease—a contemporaneous network analysis
Source: Front Med (Lausanne). 2025 Aug 6;12:1631207. doi: 10.3389/fmed.2025.1631207 (PMC12364945; doi:10.3389/fmed.2025.1631207)
Supplement: Supplementary file 1 [file Table_1.docx]

**Table S1 Edge weights for symptom networks with covariates**（due to the large size of the data, only the top 10 are shown here）

|  | **Mean** | **SD** | **CIlower** | **CIupper** |
| --- | --- | --- | --- | --- |
| nutritional deficiencies-weight loss | 0.563 | 0.036 | 0.494 | 0.637 |
| diarrhea-tenesmus | 0.342 | 0.040 | 0.268 | 0.428 |
| anxiety-depression | 0.338 | 0.049 | 0.238 | 0.434 |
| depression-disturbed sleep | 0.258 | 0.040 | 0.177 | 0.336 |
| bloody purulent stool-tenesmus | 0.237 | 0.040 | 0.159 | 0.320 |
| diarrhoea-bloody purulent stool | 0.219 | 0.046 | 0.127 | 0.313 |
| abdominal pain-abdominal distension | 0.205 | 0.053 | 0.102 | 0.313 |
| weight loss-fatigue | 0.154 | 0.040 | 0.089 | 0.249 |
| abdominal distension-disturbed sleep | 0.160 | 0.052 | 0.059 | 0.268 |
| Anxiety-disturbed sleep | 0.153 | 0.048 | 0.059 | 0.253 |
| ... | ... | ... | ... | ... |

Note: Edge weight interpretation: Regularized partial correlation coefficients (-1 to 1). Clinical meaning: Values closer to ±1 indicate stronger independent associations between symptoms after covariate adjustment. Positive values suggest co-occurrence (e.g., weight loss often accompanies nutritional deficiencies). Statistical reporting: Mean: Average bootstrap estimate (1000 samples). SD: Bootstrap standard deviation → Reflects estimate precision (lower SD = more stable). CIlower/CIupper: 95% nonparametric confidence interval → Clinically significant if CI excludes 0. Methodological note: EBICglasso algorithm (γ = 0.5) → Penalizes weak connections to enhance network interpretability. Covariates adjusted: Active disease stage, IBD duration, and non-standard treatments. Key observation: Nutritional deficiencies-weight loss shows strongest association (mean=0.563, CI[0.494-0.637])

**Table S2 Edge weights for symptom networks without covariates**（due to the large size of the data, only the top 10 are shown here）

|  | **Mean** | **SD** | **CIlower** | **CIupper** |
| --- | --- | --- | --- | --- |
| nutritional deficiencies-weight loss | 0.577 | 0.038 | 0.489 | 0.641 |
| diarrhea-tenesmus | 0.368 | 0.041 | 0.282 | 0.447 |
| anxiety-depression | 0.352 | 0.054 | 0.224 | 0.438 |
| depression-disturbed sleep | 0.270 | 0.042 | 0.167 | 0.337 |
| bloody purulent stool-tenesmus | 0.243 | 0.043 | 0.151 | 0.325 |
| diarrhoea-bloody purulent stool | 0.224 | 0.044 | 0.132 | 0.309 |
| abdominal pain-abdominal distension | 0.220 | 0.053 | 0.100 | 0.312 |
| weight loss-fatigue | 0.167 | 0.041 | 0.094 | 0.259 |
| abdominal distension-disturbed sleep | 0.165 | 0.050 | 0.059 | 0.259 |
| anxiety-disturbed sleep | 0.156 | 0.048 | 0.060 | 0.251 |
| ... | ... | ... | ... | ... |

Note: Critical comparison to Table S1: Systemic increase in mean weights (e.g., diarrhea-tenesmus: 0.368 vs 0.342) → Suggests covariates partially mediate symptom relationships. Reduced CI widths → Reflects model simplicity

**Table S3 centrality measures with covariates**

|  | Strength | Closeness | Betweenness |
| --- | --- | --- | --- |
| Diarrhea | 5.109 | 0.011 | 20.000 |
| Abdominal pain | 2.519 | 0.008 | 0.000 |
| Abdominal distension | 3.088 | 0.008 | 4.000 |
| Bloody purulent stool | 3.462 | 0.009 | 8.000 |
| Tenesmus | 4.281 | 0.010 | 22.000 |
| Perianal abscess | 1.690 | 0.006 | 2.000 |
| Anal fissure | 1.703 | 0.007 | 4.000 |
| Anal fistula | 2.272 | 0.007 | 2.000 |
| Nutritional deficiencies | 4.150 | 0.010 | 2.000 |
| Weight loss | 5.202 | 0.011 | 36.000 |
| Anaemia | 4.112 | 0.010 | 8.000 |
| Skin lesions | 2.244 | 0.007 | 0.000 |
| Oral mucosal lesions | 2.489 | 0.008 | 24.000 |
| Ocular lesions | 2.608 | 0.007 | 6.000 |
| Fatigue | 4.306 | 0.010 | 44.000 |
| Anxiety | 3.794 | 0.009 | 28.000 |
| Depression | 2.579 | 0.008 | 4.000 |
| Disturbed sleep | 2.895 | 0.008 | 16.000 |
| Stage of IBD | 4.587 | 0.011 | 50.000 |
| Years since IBD diagnosis | 2.134 | 0.008 | 2.000 |
| The type of treatment received | 2.142 | 0.007 | 6.000 |

Centrality metrics: Strength: Sum of absolute edge weights connected to a node. Higher = greater network influence. Closeness: Inverse average shortest path to other nodes. Higher = faster symptom propagation. Betweenness: Frequency of acting as a bridge between symptoms. Higher = greater control over information flow. Covariates included: Stage of IBD: Active vs. remission (assessed by gastroenterologists). Years since IBD diagnosis: Continuous variable. Type of treatment received: Medication/surgery/both/otherwise (reference: medication). Interpretation: Core symptoms: Weight loss (strength = 5.202) and Diarrhea (strength = 5.109). Covariates Stage of IBD (strength = 4.587) and Years since diagnosis (strength = 2.134) integrated as nodes. Values: Standardized centrality indices (scaled relative to maximum).

**Table S4 centrality measures without covariates**

|  | Strength | Closeness | Betweenness |
| --- | --- | --- | --- |
| Diarrhea | 4.489 | 0.013 | 24.000 |
| Abdominal pain | 2.042 | 0.009 | 0.000 |
| Abdominal distension | 2.737 | 0.010 | 4.000 |
| Bloody purulent stool | 3.122 | 0.011 | 8.000 |
| Tenesmus | 3.845 | 0.012 | 16.000 |
| Perianal abscess | 1.345 | 0.007 | 0.000 |
| Anal fissure | 1.594 | 0.008 | 8.000 |
| Anal fistula | 1.818 | 0.008 | 0.000 |
| Nutritional deficiencies | 3.560 | 0.012 | 0.000 |
| Weight loss | 4.414 | 0.013 | 28.000 |
| Anaemia | 3.683 | 0.012 | 14.000 |
| Skin lesions | 1.860 | 0.009 | 0.000 |
| Oral mucosal lesions | 2.419 | 0.010 | 28.000 |
| Ocular lesions | 1.977 | 0.008 | 4.000 |
| Fatigue | 3.818 | 0.013 | 46.000 |
| Anxiety | 3.393 | 0.011 | 24.000 |
| Depression | 2.202 | 0.009 | 2.000 |
| Disturbed sleep | 2.517 | 0.009 | 8.000 |

Note: Centrality metrics: Same as Table S3, excluding covariates. Core symptoms: Weight loss (strength = 4.414) and diarrhea (strength = 4.489) retained the highest strength centrality stability: Centrality ranking is robust to covariate adjustment (compare with Table S3).

**
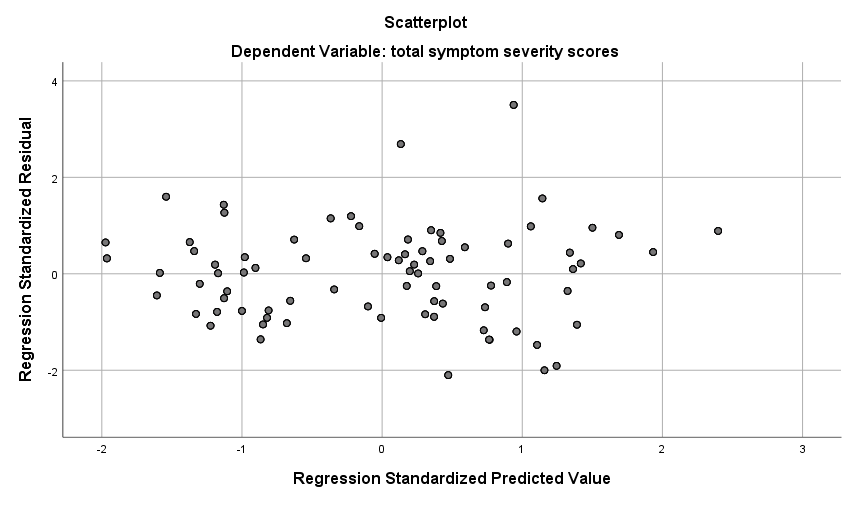
**

**Figure S1: Scatter plot of residuals from the linear regression model for overall symptom severity**

Note: Diagnostic purpose: Validates homoscedasticity assumption → Ideal pattern: Random dispersion around zero line, Red curve → Local polynomial fit; significant deviation suggests model misspecification. Clinical implication: No systematic patterns observed → Supports model validity for symptom severity prediction

**
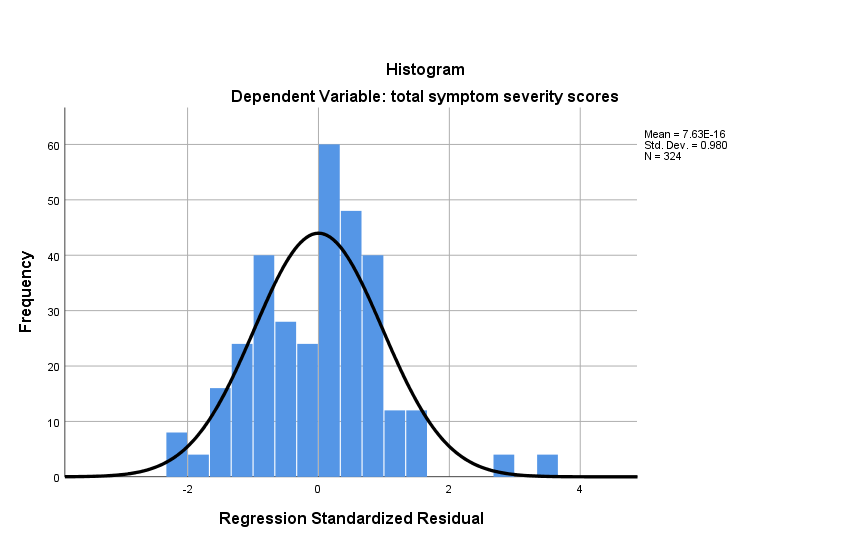
**

**Figure S2: Residual histogram of the linear regression model for overall symptom severity**

Note: Normality assessment: Gray bars → Residual frequency distribution. Black curve → Reference normal distribution. Interpretation: Symmetrical bell-shaped alignment → Satisfies normality assumption for linear regression
